# A scoping review protocol of non-verbal communication utilised in peri-interventive dental and medical procedures

**DOI:** 10.12688/hrbopenres.13373.1

**Published:** 2022-07-07

**Authors:** Paul O'Dwyer, Viveka Guzman, Emma Wallace, Frank Doyle

**Affiliations:** 1 Structured Population and Health-services Research Education PhD Programme, Royal College of Surgeons in Ireland, Dublin, Ireland; 2 Department of General Practice, Royal College of Surgeons in Ireland, University of Medicine & Health Sciences, Dublin, Ireland; 3 Department of Health Psychology, Division of Population Health Services, Royal College of Surgeons in Ireland, University of Medicine & Health Sciences, Dublin, Ireland

**Keywords:** nonverbal, dental, communication, patient

## Abstract

**Introduction**: Dental operative procedures, by their interventive nature, impede the opportunity for peri-intervention verbal communication between patients and clinicians. This can impair trust, consent, and shared decision making with the potential of adversely affecting patient dignity, and potentially resulting in suboptimal clinical outcomes.

This scoping review aims to interrogate the literature concerning non-verbal communication methods used in dental and medical practices during peri-interventive procedures, in aiding communication between clinician and patient. We will also ascertain how these align with the Limited Capacity Model of Motivated Mediated Message Processing (LC4MP) communication theory.

**Methods**: The framework proposed by the Joanna Briggs Institute and the Preferred Reporting Items for Systematic Reviews and Meta-analysis extension for scoping reviews, will be used to guide this scoping review and reporting methodology. Selected electronic databases (Medline, Embase, Cochrane Library and Scopus), PsychInfo, CINAHL and grey literature sources will be searched.

Inclusion criteria are: articles written in the English language, publications between 2000 and 2020, peer-reviewed empirical studies, with either qualitative or quantitative data, mixed methods, reviews, book chapters and grey literature with a principal focus on non-verbal communication in the healthcare setting.

A narrative synthesis will be conducted, with results reported according to elements of LCM4P theory: cognitive load, motivated messaging, message processing and memory.

**Conclusion**: This scoping review will contribute to our methodological and theoretical understanding of the use of non-verbal communication strategies in clinical settings.

## Introduction

Dental operative procedures, by their interventive nature, impede the opportunity for peri-intervention verbal communication between patients and clinicians. While good communication is enshrined in policy ^1^, expected in clinical standards ^2^, and advocated as best practice ^3^, there is a risk that such impaired communication could erode or impair trust, consent, shared decision making and adversely impact on patient dignity and clinical outcomes ^4^. Therefore, a focus on enhancing peri-interventive communication could be beneficial for patients, clinicians and the healthcare system.

Internationally, a recent adult Dental Health Survey in the United Kingdom (UK) reported that 11% of patients complained of *“…not being involved in decisions about dental care or treatment”* ^5^. Patients felt less satisfied with their involvement in decision making for treatment planning and this impacted on their clinical outcomes. Elsewhere in the literature, certain key areas need to be addressed for study which include intra-operative communication and active involvement of the patient ^6^. Active intra-operative communication would include feedback from the operator/clinician. Poor interpersonal communication was identified as the most common barrier to adherence with dental treatment for female caregivers of children in a study in the United States (US) ^7^. The literature also advocates increasing focus on communication skills in dental students, as an integral part of undergraduate curricula ^8^. In Jamaica, Jamaican Sign Language has been made compulsory in the education of dental auxiliaries to reduce barriers to attendance for the deaf community and to safeguard dignity and consent in that jurisdiction ^9^. 

Suboptimal clinical outcomes in dentistry can lead to litigation ^10^. Barriers to effective communication, particularly during intervention, have been identified by the medical indemnifier (Medical Protection Society) as a leading cause of litigation with the majority of negligence cases not related to the clinical quality of care but rather triggered by inadequate communication ^11^. This was underlined by the subsequent UK National Health Service publication *“Much More Than Words”,* which advocates non-verbal communication as a useful tool in patient communication ^12^. This is further supported by a US national Dentist-Patient survey, which reports “... *routine use of all of the communication techniques is low among dentists, including some techniques thought to be most effective with patients with low literacy skills*”. These techniques included non-verbal communication with patients (such as hand-signalling or maintaining eye-contact) ^13^.

Communication theory can guide and optimise communication strategies ^14^. It is argued that communication theories should accomplish three goals: (i) be inclusive of a wide range of phenomena; (ii) strive for explanatory and predictive power; and (iii) strive for practical utility in real-world communication scenarios ^15^. One such theory is the Limited Capacity Model of Motivated Mediated Message Processing (LC4MP) ^16^. This theory attempts to elucidate understanding of the dynamic interactions between mediated messages and the human information processing system, particularly in the healthcare setting. It provides a theoretical and methodological framework to investigate an individual’s interaction with communication phenomena in real time. LC4MP captures the following three domains: cognitive load (how the limited capacity human information processing system is impacted by message characteristics) motivated messaging (multidimensional emotional responses to message processing) and memory. This theory can provide a structure to appraise the communication strategies and highlight any pertinent gaps. LC4MP has been extensively used in research for medical messaging regarding behavioural modification, e.g. smoking cessation ^17^. This theory will be used in this research to support a robust investigation of real-time patient interaction and the effects of using a nonverbal communication system. 

To date, no review of non-verbal clinical interactions has been conducted using this theory. Therefore, the aim of this scoping review is to assess the literature for non-verbal communication in peri-operative clinical settings. Specifically, we will:

(1) Identify the use of non-verbal communication systems in dental and medical clinical settings
(2) Ascertain reported communication difficulties and facilitators in peri-interventive procedures in dental and medical settings
(3) Determine the theoretical underpinnings of reported non-verbal interventions, according to LC4MP theory
(4) Identify typical methodological aspects, such as outcome measures.

Ultimately, the findings will inform intervention development for non-verbal peri-intervention communication strategy in dentistry.

## Methods

### Scoping review framework

This scoping review will follow the framework proposed by the Joanna Briggs Institute (JBI) ^18^, and specifically it will follow the method described by O’Brien *et al*. ^19^. While the overall conduct of the scoping review is informed by the JBI framework, the Preferred Reporting Items for Systematic Reviews and Meta-analysis extension for scoping reviews (PRISMA-ScR) ^20^ will be used to guide the reporting of the scoping review.

### Stage 1: Identifying the research question

Overarching question: What non-verbal methods of communication currently exist/are used in dental/medical clinical settings in both primary and secondary care, what are the theoretical underpinnings of these communication methods, and what methods are used to evaluate these communication methods?

Objectives: 

a. Describe the methods, definitions and underpinning theories of non-verbal communication currently in use in peri-interventive scenarios

b. Outline the development and methodological evaluation of these systems.

### Stage 2: Identifying the relevant studies

**Table 1.** Eligibility criteria for scoping review.

Inclusion criteria	Rationale
• Non-verbal peri-interventive communication systems used in clinical settings	Any non-verbal system used within the clinical setting to aid communication could be useful to inform intervention development
• For the purposes of this review, we define non-verbal communication as *an exchange of information expressed in a range of physical movements, gestures or facial expressions between patient and clinician*	

### Search strategy

The search strategy will identify both published and grey literature and will follow a three-step strategy, as per JBI.

The **first step** is a limited search. This will include the searching of two appropriate online databases, in this case Medline and Embase. Analysis will then be conducted on the titles and abstracts of retrieved papers. An analysis will also be undertaken of the index terms used to describe the articles The data extracted will be represented in a tabular and descriptive format that aligns with the review’s objective.

The **second step** will use all identified key words and index terms to perform a second search (PO’D and VG) of the following databases: Medline, Embase, Cochrane Library, Scopus, PsycInfo, and CINAHL to identify peer reviewed research papers relevant to our aim. This step will be conducted in collaboration with an information specialist.

The **third step** will be to use reference lists of included articles to search for additional sources.

The grey literature search will be completed by searching ‘Open Grey’ using a similar methodology as above.

The final included studies will be stored and combined using a reference management software package (EndNote) and duplicates will be removed.

### Stage 3: Study selection

Titles and abstracts will be screened (PO’D and VG) for inclusion against the inclusion criteria for the review ( Table 1). Full text articles of those that appear to meet the inclusion criteria will then be retrieved and screened against the inclusion criteria. For articles that cannot be retrieved, we will contact the authors directly. Those articles that fulfil all the inclusion criteria will be included in the review. At this stage we will also use the ‘cited by’ function in Google Scholar to obtain other relevant articles.

Two reviewers (PO’D, VG) will initially work independently and then come together to compare results. Any discrepancies will be resolved by consensus and if consensus is not reached then they will be referred to a third reviewer (FD).

Studies that do not meet the inclusion criteria will be excluded. Reasons for the exclusion will be provided in an Appendix in the final draft featuring a flow diagram. These will be grouped according to common reasons for exclusion.

The final search results will be displayed in a PRISMA flow diagram, alongside a narrative description of the process.

### Stage 4: Charting the data

This scoping review is designed to identify the range of evidence available and represent this as a mapping of the identified data, without the act of synthesis or with particular reference to methodological quality of relevant studies ^21^. 

For this scoping review data extraction will adapt the standardised template from the JBI methodology guidance for scoping reviews. 

Data extracted here will be subdivided into categories: author, year, setting, aim, sample description and size, study design, description of intervention (including elements of LC4MP: cognitive load, motivation and memory), outcome measures and main results.

### Stage 5: Collating, summarising and reporting of results

Results will be reported using the PRISMA-SCR guidelines. Each research question will be reported and presented in a tabular form and as a narrative summary. This narrative description will be used to synthesise the study findings based on themes that are generated from the extracted data, using the LCM4P framework to further delineate results.

## Discussion

This scoping review will be the first to utilise communication theory to provide a structure and overview of the literature of non-verbal peri-interventive communication in dental and medical practice. This can contribute to the evidence base to support the use of non-verbal communication by healthcare professionals and patients to benefit patient care. The findings of this scoping review will inform future research on the evaluation and implementation of non-verbal communication in clinical settings.

## Dissemination

We intend to disseminate the results through publication in a peer-reviewed journal and conference presentations.

## Study status 

The study is about to commence (June 2022), with expected completion by December 2022.

## Ethics statement 

Ethical approval is not required for this scoping review.

## Data availability

No data are associated with this article.
