# Supplementary material for: A scoping review protocol of non-verbal communication utilised in peri-interventive dental and medical procedures
Source: HRB Open Res. 2025 Mar 3;5:49. Originally published 2022 Jul 7. [Version 2] doi: 10.12688/hrbopenres.13373.2 (PMC11920688; doi:10.12688/hrbopenres.13373.2)
Supplement: Peer review history

# Reviewer report: Reviewer response for version 2

**DOI:** 10.21956/hrbopenres.15477.r46270

**Published:** 2025-03-18

**Authors:** Esther Lydie Wanko Keutchafo

**Affiliations:** 1 University of KwaZulu-Natal, Durban, South Africa

Thank you for satisfactorily addressing all my comments. The manuscript is greatly improved.

Is the study design appropriate for the research question?

Yes

Is the rationale for, and objectives of, the study clearly described?

Yes

Are sufficient details of the methods provided to allow replication by others?

Partly

Are the datasets clearly presented in a useable and accessible format?

Not applicable

Reviewer Expertise:

NA

I confirm that I have read this submission and believe that I have an appropriate level of expertise to confirm that it is of an acceptable scientific standard.

# Reviewer report: Reviewer response for version 2

**DOI:** 10.21956/hrbopenres.15477.r46269

**Published:** 2025-03-06

**Authors:** Abukari Kwame

**Affiliations:** 1 University of Saskatchewan, Saskatoon, Canada

I have read the manuscript and found that the authors successfully implemented my suggestions.

 They have also addressed all the comments satisfactorily.

 I have no further comments.

Is the study design appropriate for the research question?

Yes

Is the rationale for, and objectives of, the study clearly described?

Yes

Are sufficient details of the methods provided to allow replication by others?

Yes

Are the datasets clearly presented in a useable and accessible format?

Not applicable

Reviewer Expertise:

NA

I confirm that I have read this submission and believe that I have an appropriate level of expertise to confirm that it is of an acceptable scientific standard.

# Reviewer report: Reviewer response for version 1

**DOI:** 10.21956/hrbopenres.14566.r34284

**Published:** 2023-11-28

**Authors:** Esther Lydie Wanko Keutchafo

**Affiliations:** 1 University of KwaZulu-Natal, Durban, South Africa

This manuscript is a scoping review protocol on non-verbal communication strategies used in peri-interventive dental and medical procedures. A background section has been provided to justify why the intended review is warranted. Furthermore, a step-by-step methodology has been provided on how the review will be conducted.

 However, the following issues must be addressed: 

 1. The authors must define peri-interventive dental and medical procedures as well as non-verbal communication in the background section.

 2. The authors outlined their specific aims which do not correspond to their questions nor objectives. How will they achieve those aims? Please match the research questions and objectives to your specific aims.

 3. The table of eligibility criteria must include one column for inclusion criteria and one for exclusion with some examples based on the PCC (Population, Concept, Context) framework if it is the PCC framework you are used to frame your research question. As such, the table presented does not inform us about the criteria.

 4. Please provide a rationale for language limitation as well as date limitation in the methodology, especially because you stated in the abstract that it will be articles in English from 2000 to 2020 when we are already in 2023. 

 5. Did you conduct a preliminary search to identify the possibility of finding relevant articles? If yes, what keywords or Mesh terms did you use to search for articles? If no, please provide a table of a preliminary search with keywords used, filters, results, and time. This is to ascertain that you won't conduct a review that will yield no result.

Is the study design appropriate for the research question?

Yes

Is the rationale for, and objectives of, the study clearly described?

Yes

Are sufficient details of the methods provided to allow replication by others?

Partly

Are the datasets clearly presented in a useable and accessible format?

Not applicable

Reviewer Expertise:

Non-verbal communication, clinical nursing care

I confirm that I have read this submission and believe that I have an appropriate level of expertise to confirm that it is of an acceptable scientific standard, however I have significant reservations, as outlined above.

# Response

**Published:** 2025-02-07

**Authors:** Paul O Dwyer

**Affiliations:** Royal College of Surgeons in Ireland, Ireland

PEER REVIEW Responses to Scoping Protocol

**REVIEW 1**

 APPROVED WITH RESERVATIONS

 info_outline

**Review of “A scoping review protocol of non-verbal communication utilised in peri-interventive dental and medical procedures” This manuscript is a scoping review protocol that outlines and justifies the process to be undertaken to scope and review the literature on non-verbal communication strategies used in peri-interventive dental and medical procedures. The protocol outlines the research questions to be explored, the methodology to be employed, and the possible outcomes to be achieved. A detailed background section has been provided to outline a significant gap in the literature and to justify why the intended review is warranted. Furthermore, a clear step-by-step methodology has been provided on how the review will be conducted. However, a few issues must be addressed before the protocol can be indexed. I recommend that the protocol may be accepted for indexing after the following issues are addressed.**

 We thank the reviewer for the overall positive appraisal.

**Background Section**

**1. Given that the scoping review centers on communication in peri-intervention dental and medical settings, the author/s must explain what peri-interventive procedure/s is/are and why non-verbal communication is crucial in that setting.**

 Peri-interventive clinical interactions/interventive procedures are provided with/without the use of local anaesthetic (e.g. restorative procedure in dentistry). For interventive dental procedures, where the patient’s mouth is full (with instruments), there is a risk of impaired communication and potential erosion of trust, consent and shared decision making – as verbal communication can be challenged or absent completely. We have now included this definition in the protocol:

*“Peri-interventive procedures are defined as patient-centred, multidisciplinary and integrated medical care for patients from the onset of procedural preparation until full recovery *( **1**). Verbal communication can be challenged or absent completely while the patient’s mouth is treated with instruments which exacerbates the risk of impaired communication and potential erosion of trust, consent, and shared decision making. In this context, non-verbal communication is a viable option to alleviate these issues.”

**Methodology Section**

**1. Scoping review framework: Although the author/s made references to standard reporting procedures for a scoping review, the main methodology for any scoping review, based on scholarly consensus, is that of Hilary Arksey & Lisa O'Malley (2005) Scoping studies: towards a methodological framework. I wonder why the author/s is/are not following the methodology of scoping review as outlined in Arksey and O’Malley (2005)**

 Thank you for this recommendation. The scoping review protocol does now adhere to framework methodology of Arksey and O’Malley **(21)**  including the following stages: identifying the research question (Page 4); identifying relevant studies (Page 5); study selection (Page 6); charting the data; and collating, summarizing, and reporting the results (Page 7) . Arksey and O’Malley do suggest an Optional Consultation 6 ^th^ Stage. This is not included here.

 To enhance clarity, we have added the following to the protocol:

 “This protocol follows the Arksey & O’Malley **(21)** methodological recommendations, with the exception of the optional consultation stage.”

**2. Search strategy: What keywords/phrases will be used to search for articles? The author/s must identify and state some keywords to be used when searching databases as part of this protocol.**

 The second stage for the framework methodology as outlined by Arksey **(21)** involves the search strategy via identifying relevant studies. We have now included examples of the search terms within the methods, and a comprehensive list of exact terms and combination in Appendix A.

**3. Study selection: Unless the review is going to be a review of reviews, is it not advisable to include reviews in a scoping review study? The authors mention in the abstract that reviews will be included in the study. Empirical studies and grey literature can be included but not reviews. The findings of reviews can conflate that of primary research results. If the author/s still want/s to include reviews in the proposed scoping review, then they must justify that.**

 Thank you for spotting this error. Reviews will not be included and the abstract has been amended to reflect this change.

**4. Also, why will the scoping review include only studies published between 2000 and 2022? Any justification for this limitation?**

 Preliminary literature searches could not find any relevant data before this date and so we decided to formally review the literature from 2000 onwards.

**5. Stage 5: Using the LC4MP theory in the proposed scoping review will be critical and novel. However, will the author/s be including primary studies that utilize the theory? In other words, how will the LC4MP theory guide the analysis of the extracted data from included studies? Also, how will the thematic analysis be undertaken since the author/s indicate/s that themes will be generated from the extracted data?**

 We will not be conducting a thematic analysis per se, so this has been modified. We have clarified the procedure as follows:

 “Data extracted here will be subdivided into categories: author, year, setting, aim, sample description and size, study design, description of intervention (including elements of LC4MP: cognitive load, motivation and memory), outcome measures and main results. Any statements or elements pertaining to the authors consideration of cognitive load, motivation or memory, the components of LC4MP, will be specifically extracted from the included studies and included in tabular form as part of the overall results.”

**6. Stage 6: Arksey and O’Malley (2005) scoping review framework includes stage 6, where authors must engage experts in the specific field of the review to gain further knowledge about the topic under review. This protocol does not say anything about that critical stage in scoping reviews. The author/s must justify why this step will not be implemented in their review.**

 Thank you for this suggestion. The proposed Stage 6 consultation exercise in the framework is optional **(21). **This process is not considered as part of the PRIMSA-ScR **(23)** recommendation and we have decided not to include it in this review.

 · Is the rationale for, and objectives of, the study clearly described?

 Yes

 · Is the study design appropriate for the research question?

 Yes

 · Are sufficient details of the methods provided to allow replication by others?

 Yes

 · Are the datasets clearly presented in a useable and accessible format?

 Not applicable

**REVIEW 2**

**APPROVED WITH RESERVATIONS**

**This manuscript is a scoping review protocol on non-verbal communication strategies used in peri-interventive dental and medical procedures. A background section has been provided to justify why the intended review is warranted. Furthermore, a step-by-step methodology has been provided on how the review will be conducted.**

**However, the following issues must be addressed:**

**1. The authors must define peri-interventive dental and medical procedures as well as non-verbal communication in the background section.**

 We have now provided the following definitions in the Background:

*“Peri-interventive procedures are defined as patient-centred, multidisciplinary and integrated medical care for patients from the onset of procedural preparation until full recovery”*( **1**). Verbal communication can be challenged or absent completely while the patient’s mouth is treated with instruments which exacerbates the risk of impaired communication and potential erosion of trust, consent, and shared decision making. In this context, non-verbal communication is a viable option to alleviate these issues.

 “Nonverbal communication is defined as nonlinguistic, informative aspects of behaviour and appearance, including head and body features or movements, touch, interpersonal distance, and paralanguage (4).”

**2. The authors outlined their specific aims which do not correspond to their questions nor objectives. How will they achieve those aims? Please match the research questions and objectives to your specific aims.**

 The aims here are to ascertain the level to which non-verbal communication currently exists within the peri-operative visit. By conducting this scoping review, the literature will be interrogated to examine this. We have now modified and aligned the Research Question with the Aim and Objectives. We provide the following table here to illustrate the alignment, and have made the relevant adjustments in the document:

 Research question

 Aim

 Objectives

 Stage 1: Identifying the research question

 Overarching question: What non-verbal methods of communication

 currently exist/are used in dental/medical clinical

 settings in both primary and secondary care, what are the theoretical

 underpinnings of these communication methods, and what

 methods are used to evaluate these communication methods?

 Abstract

 This scoping review aims to interrogate the literature concerning nonverbal

 communication methods used in dental and medical practices

 during peri-interventive procedures, in aiding communication

 between clinician and patient. We will also ascertain how these align

 with the Limited Capacity Model of Motivated Mediated Message

 Processing (LC4MP) communication theory.

 (1) Identify the use of non-verbal communication systems

 in dental and medical clinical settings

 (2) Ascertain reported communication difficulties and

 facilitators in peri-interventive procedures in dental

 and medical settings

 (3) Determine the theoretical underpinnings of reported

 non-verbal interventions, according to LC4MP theory

 (4) Identify typical methodological aspects, such as outcome

 measures.

**3. The table of eligibility criteria must include one column for inclusion criteria and one for exclusion with some examples based on the PCC (Population, Concept, Context) framework if it is the PCC framework you are used to frame your research question. As such, the table presented does not inform us about the criteria.**

 The table is now amended to reflect this.

**Table 2:** *Eligibility criteria*

**Inclusion Criteria**

**Exclusion Criteria**

**Population**

**Concept**

**Context**

**Population**

**Concept**

**Context**

 >18 Years

 Non-Verbal Communication

 Clinical Setting

 <18 Years

 Verbal Communication

 Non Clinical Setting

**4. Please provide a rationale for language limitation as well as date limitation in the methodology, especially because you stated in the abstract that it will be articles in English from 2000 to 2020 when we are already in 2023.**

 Please see response above for the rationale for excluding literature pre-2000. The investigators have no resources to examine non-English articles. We will now update the search to include literature to 2025.

**5. Did you conduct a preliminary search to identify the possibility of finding relevant articles? If yes, what keywords or Mesh terms did you use to search for articles? If no, please provide a table of a preliminary search with keywords used, filters, results, and time. This is to ascertain that you won't conduct a review that will yield no result.**

 Yes – a preliminary search was conducted. This preliminary search utilised key terms nonverbal communication, sign language , communication barriers and perioperative care. This yielded some promising literature and we decided to proceed.

 This was the MESH search used:

 ((((((nonverbal communication[Title/Abstract]) OR (nonverbal signal[Title/Abstract])) OR (gesture[Title/Abstract])) OR (sign language[Title/Abstract])) OR (communication barriers[Title/Abstract])) OR ("Nonverbal Communication"[Mesh])) OR ("Communication Barriers"[Mesh])) OR ("Sign Language"[Mesh])) AND (("Operating Rooms"[Mesh]) OR ("Perioperative Care"[Mesh])) AND dent*

- **Is the rationale for, and objectives of, the study clearly described?**

Yes

- **Is the study design appropriate for the research question?**

Yes

- **Are sufficient details of the methods provided to allow replication by others?**

Partly

- **Are the datasets clearly presented in a useable and accessible format?**

Not applicable

# Reviewer report: Reviewer response for version 1

**DOI:** 10.21956/hrbopenres.14566.r34286

**Published:** 2023-06-15

**Authors:** Abukari Kwame

**Affiliations:** 1 University of Saskatchewan, Saskatoon, Canada

**Review of “A scoping review protocol of non-verbal communication utilised in peri-interventive dental and medical procedures”**

 This manuscript is a scoping review protocol that outlines and justifies the process to be undertaken to scope and review the literature on non-verbal communication strategies used in peri-interventive dental and medical procedures. The protocol outlines the research questions to be explored, the methodology to be employed, and the possible outcomes to be achieved.

 A detailed background section has been provided to outline a significant gap in the literature and to justify why the intended review is warranted. Furthermore, a clear step-by-step methodology has been provided on how the review will be conducted. However, a few issues must be addressed before the protocol can be indexed. I recommend that the protocol may be accepted for indexing after the following issues are addressed.

**Background Section**

1. Given that the scoping review centers on communication in peri-intervention dental and medical settings, the author/s must explain what peri-interventive procedure/s is/are and why non-verbal communication is crucial in that setting.

**Methodology Section**

1. **Scoping review framework**: Although the author/s made references to standard reporting procedures for a scoping review, the main methodology for any scoping review, based on scholarly consensus, is that of Hilary Arksey & Lisa O'Malley (2005) Scoping studies: towards a methodological framework. I wonder why the author/s is/are not following the methodology of scoping review as outlined in Arksey and O’Malley (2005) ^1^.
2. **Search strategy**: What keywords/phrases will be used to search for articles? The author/s must identify and state some keywords to be used when searching databases as part of this protocol.
3. **Study selection**: Unless the review is going to be a review of reviews, is it not advisable to include reviews in a scoping review study? The authors mention in the abstract that reviews will be included in the study. Empirical studies and grey literature can be included but not reviews. The findings of reviews can conflate that of primary research results. If the author/s still want/s to include reviews in the proposed scoping review, then they must justify that.
4. Also, why will the scoping review include only studies published between 2000 and 2022? Any justification for this limitation?
5. **Stage 5:** Using the LC4MP theory in the proposed scoping review will be critical and novel. However, will the author/s be including primary studies that utilize the theory? In other words, how will the LC4MP theory guide the analysis of the extracted data from included studies? Also, how will the thematic analysis be undertaken since the author/s indicate/s that themes will be generated from the extracted data?
6. **Stage 6:** Arksey and O’Malley (2005) scoping review framework includes stage 6, where authors must engage experts in the specific field of the review to gain further knowledge about the topic under review. This protocol does not say anything about that critical stage in scoping reviews. The author/s must justify why this step will not be implemented in their review.

Is the study design appropriate for the research question?

Yes

Is the rationale for, and objectives of, the study clearly described?

Yes

Are sufficient details of the methods provided to allow replication by others?

Yes

Are the datasets clearly presented in a useable and accessible format?

Not applicable

Reviewer Expertise:

Health communication, patient-provider interactions, medical anthropology, language acquisition, qualitative research methods, and Indigenous research methodology

I confirm that I have read this submission and believe that I have an appropriate level of expertise to confirm that it is of an acceptable scientific standard, however I have significant reservations, as outlined above.

## References

[1] : Medical mistrust among women with intersecting marginalized identities: a scoping review. *Ethn Health* .2022;27(8) : 10.1080/13557858.2021.1990220 1733-1751 10.1080/13557858.2021.1990220 34647832
